# Elevated urine albumin creatinine ratio increases cardiovascular mortality in coronary artery disease patients with or without type 2 diabetes mellitus: a multicenter retrospective study

**DOI:** 10.1186/s12933-023-01907-3

**Published:** 2023-08-10

**Authors:** Xueqin Lin, Wei Song, Yang Zhou, Yuwei Gao, Yani Wang, Yun Wang, Yuchen Liu, Lin Deng, Yin Liao, Bo Wu, Shiqun Chen, Liling Chen, Yong Fang

**Affiliations:** 1 Longyan First Affiliated Hospital of Fujian Medical University, Longyan, 364000 China; 2 Department of Cardiology, Guangdong Provincial People’s Hospital, Guangdong Cardiovascular Institute, Guangdong Academy of Medical Sciences, Guangzhou, 510080 China; 3 Department of Guangdong Provincial Key Laboratory of Coronary Heart Disease Prevention, Guangdong Provincial People’s Hospital, Guangdong Cardiovascular Institute, Guangdong Academy of Medical Sciences, Guangzhou, 510080 China; 4 Jinan university, Zhuhai people’s hospital, Guangzhou, 510100 China; 5 Global Health Research Center, Guangdong Provincial People’s Hospital, Guangdong Academy of Medical Science, Guangzhou, 510100 China

**Keywords:** Coronary artery disease, Urine albumin creatinine ratio, Diabetes mellitus, Mortality

## Abstract

**Background:**

Albuminuria has been suggested as an atherosclerotic risk factor among the general population. However, whether this association will be amplified in patients with coronary artery disease (CAD) is unknown. It is also unknown whether diabetes mellitus confounds the association. We aim to analyse the prognosis of elevated urine albumin creatinine ratio (uACR) in the CAD population with or without type 2 diabetes mellitus (T2DM).

**Methods:**

This multi-center registry cohort study included 5,960 patients with CAD. Patients were divided into T2DM and non-T2DM group, and baseline uACR levels were assessed on three grades (low: uACR < 10 mg/g, middle: 10 mg/g ≤ uACR < 30 mg/g, and high: uACR ≥ 30 mg/g). The study endpoints were cardiovascular mortality and all-cause mortality.

**Results:**

During the median follow-up of 2.2 [1.2–3.1] years, 310 (5.2%) patients died, of which 236 (4.0%) patients died of cardiovascular disease. CAD patients with elevated uACR had a higher risk of cardiovascular mortality (middle: HR, 2.32; high: HR, 3.22) than those with low uACR, as well as all-cause mortality. Elevated uACR increased nearly 1.5-fold risk of cardiovascular mortality (middle: HR, 2.33; high: HR, 2.34) among patients without T2DM, and increased 1.5- fold to 3- fold risk of cardiovascular mortality in T2DM patients (middle: HR, 2.49; high: HR, 3.98).

**Conclusions:**

Even mildly increased uACR could increase the risk of cardiovascular mortality in patients with CAD, especially when combined with T2DM.

**Supplementary Information:**

The online version contains supplementary material available at 10.1186/s12933-023-01907-3.

## Introduction

According to the Global Burden of Disease Study, coronary artery disease (CAD) is a major cause of morbidity and mortality globally [1]. Once patients with CAD combine with type 2 diabetes mellitus (T2DM), it can directly affect the myocardium, resulting in microvascular dysfunction, myocardial fibrosis, and left ventricular hypertrophy [2].

Urine albumin creatinine ratio (uACR) is an indicator to predict kidney disease and reflect kidney function. Kidney Disease Outcomes Quality Initiative (KDOQI) guildline clearly reports that patients with uACR > 30 mg/g have significantly increased renal progression and cardiovascular risk, leading to multiple pathophysiological processes, including endothelial dysfunction, diffuse vascular damage, systemic inflammation, renal damage related to altered glomerular hemodynamics, and abnormal tubular function [3, 4]. In addition, patients with T2DM combine with uACR elevation should be managed comprehensively to reduce the occurrence of cardiovascular and renal events. Although the ADA guideline for Standards of Care in Diabetes suggests that uACR should be controlled below 30 mg/g [5, 6], there are still plenty of research pointing out that the risk of major adverse cardiovascular events (MACE) and mortality for patients will increase even uACR is elevated within the safe range (< 30 mg/g) [7–10]. In addition, in high-risk CAD patients with T2DM, few studies focus on the effect of a slight increase in uACR levels on prognosis, and whether it is affected by diabetic status.

Therefore, we aim to explore whether slightly elevated uACR increases the risk of all-cause and cardiovascular mortality in CAD patients and whether this risk is further aggravated when DM is combined. We hope that this study will provide a basis for early detection and treatment of CAD patients with low uACR level, as well as the selection of clinical treatment.

## Methods

### Study design and population

**Fig. 1:**
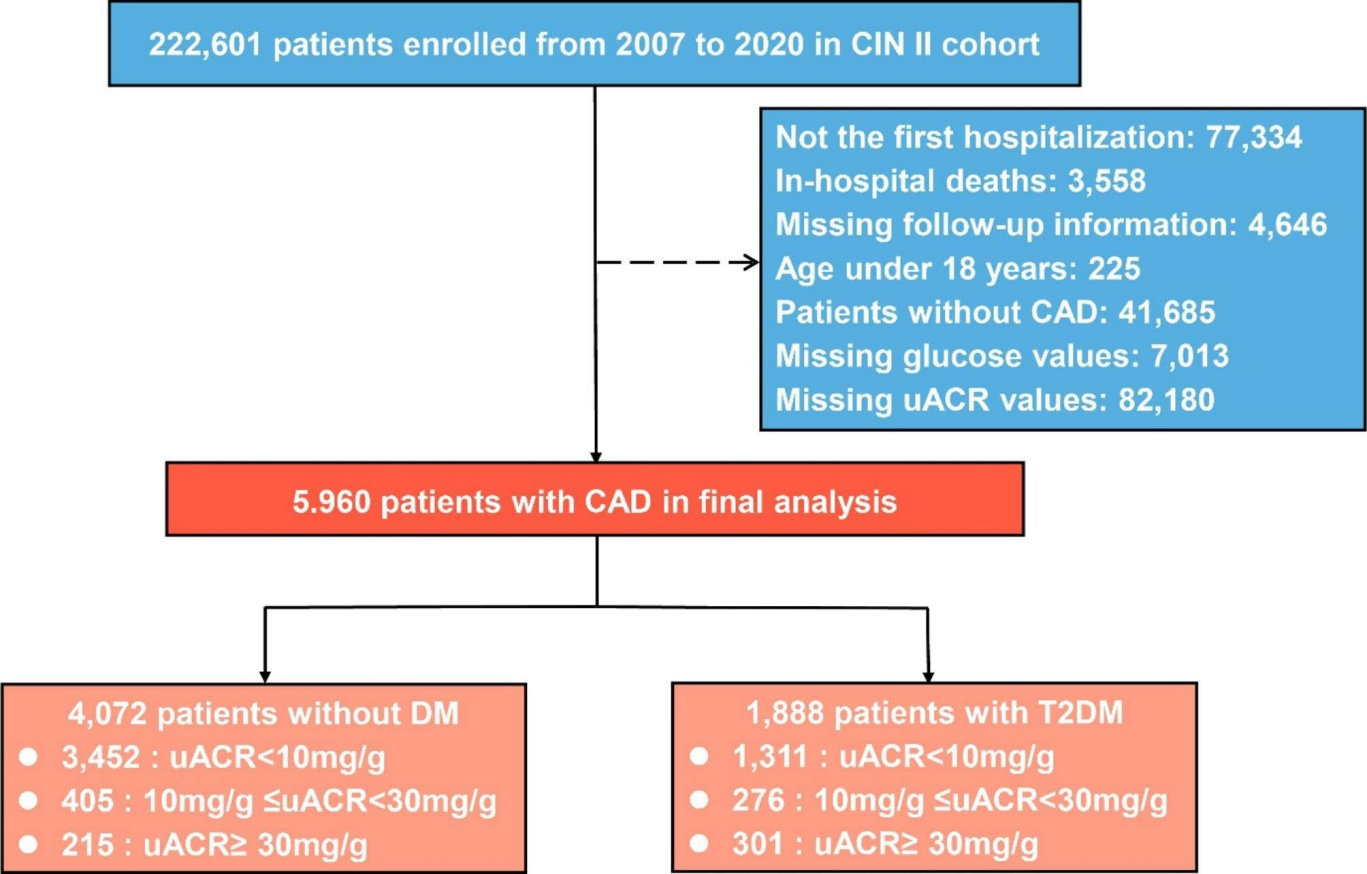
Flow diagram Abbreviations: CAD: Coronary artery disease; uACR: Urine albumin creatinine ratio; T2DM: Type 2 diabetes mellitus

This multi-center, retrospective study was based on the registry of Cardiorenal ImprovemeNt II (CIN-II, NCT05050877) cohort from January 2000 to December 2020 in five south Chinese regional central tertiary teaching hospitals [11]. Patients not hospitalized for the first time were excluded, as well as patients without discharge status reporting. Patients without records of uACR and DM status were excluded. Finally, 5,960 participants with CAD in CIN-II were included in our study (see Fig. 1).

The study protocol was approved by the Guangdong Provincial People’s Hospital ethics committee (No. GDREC2019-555 H-2), all participating sites received institutional review board approval from their own ethics committees, and the study was performed according to the declaration of Helsinki.

### Data collection

The follow-up information was obtained by matching the survival information of patients with Centers for Disease Control and Prevention. The hospital patient data was collected from the Electronic Clinical Management System (ECMS). After admission for CAD diagnosis, patients’ urine samples were collected the next early morning, and the midstream urine was collected. Urinary albumin excretion was measured on spot urine specimens by immunoturbidimetry with goat anti-human albumin antiserum with a coefficient of variation < 10%. Urinary creatinine was measured by using a colorimetric dye-binding technique based on its reaction with picric acid with a coefficient of variation < 6% [12]. Both tests were performed on a laboratory analyser with standard reagents (Beckman Coulter AU5800, Beckman Coulter, Inc., Brea, CA). UACR values were calculated as mg albumin/g creatinine.

### Outcomes and definitions

The main outcome of the study were all-cause mortality and cardiovascular mortality. T2DM was accessed according to the discharge diagnosis. CAD was confirmed by CAG and discriminated according to the 10th Revision Codes of the International Classification of Diseases. Hypertension (HT) was defined according to the 10th Revision Codes of the International Classification of Diseases (I10.xxx-I12.xxx, I15.xxx and I67.400). Congestive heart failure (CHF) was defined as the discharge diagnosis and New York Heart Association class > 2 or Killip class > 1 [13]. Chronic kidney disease (CKD) was defined as the discharge diagnosis and estimated glomerular filtration rate (eGFR) ≤ 60 mL/min/1.73 m2 by Chronic Kidney Diseases Epidemiology Collaboration equation (CKD-EPI) [14]. Anemia was defined using World Health Organization criteria: baseline hematocrit value < 39% for men and < 36% for women [15].

### Statistical analysis

The study included 5,960 participants with CAD. These patients were divided into 2 groups according to their level of DM status: patients without T2DM and patients with T2DM. Baseline uACR levels were divided into three groups: uACR < 10 mg/g (low); 10 mg/g ≤ uACR < 30 mg/g (middle); uACR ≥ 30 mg/g (high) [16, 17].

The continuous variables were summarised as mean (SD) or median [interquartile ranges (IQRs)], and categorical variables were presented as counts and proportions. Characteristics were compared between groups using ANOVA, Kruskal Wallis tests and χ^2^ tests as appropriate. Kaplan-Meier method (KM) and log-rank test was used to analyse cumulative hazard of all-cause mortality. In addition, cumulative incidence function (CIF) was used to calculate cardiovascular mortality, and Gray’s test was used to investigate their group differences. Restricted cubic spline was used to show the association between uACR levels and mortality among groups. Cox regression and Fine-Gray competing risks models were used to assess the association between uACR level and the risk of all-cause mortality and cardiovascular mortality, respectively. Characteristic variables with significant baseline differences or clinical significance were used as candidate predictors in the multivariate regression model. Model 1 was unadjusted, model 2 was adjusted for age and gender, and model 3 was adjusted for age, gender, smoking history, acute myocardial infarction, hypertension, chronic kidney disease, congestive heart failure, anemia, T2DM, low density lipoprotein cholesterol, high density lipoprotein cholesterol and ACEI/ARB. In addition, we assessed the influence of elevated uACR on all-cause and cardiovascular mortality in patients with or without T2DM, and patients with or without CHF (see supplement Fig. 1). Adjusted hazards ratios with 95% confidence intervals were calculated for each variable. All statistical analyses were performed using R software (version 4.2.2). A two-sided P-value < 0.05 indicated significance for all analyses.

## Results

### Baseline characteristics

We included 5,960 patients with CAD [mean age 62.8 ± 10.4 years, 24.1% females] in our study. Totally 1,888 patients were diagnosed as T2DM, 4,763 patients were at low uACR level (< 10 mg/g), 681 patients were at middle uACR level (10–30 mg/g) and 516 patients had high uACR level (≥ 30 mg/g).

Compared with patients without T2DM, those combined with T2DM were older (63.2 vs. 62.6 years, P = 0.03), had high rate of hypertension (69.5% vs. 57.1%, P < 0.001), congestive heart failure (15.4% vs. 11.7%, P < 0.001), chronic kidney disease (22.8% vs. 13.3%, P < 0.001), stroke (9.7% vs. 6.8%, P < 0.001), hyperlipemia (74.2% vs. 64.3%, P < 0.001), and anemia (40.5% vs. 30.2%, P < 0.001). In addition, with the increase of patients’ uACR level, the incidence of comorbidities also increased significantly, such as acute myocardial infarction (14.6% vs. 20.7% vs. 23.4%, P < 0.001), hypertension (58.2% vs. 65.5% vs. 81.2%, P < 0.001), T2DM (27.5% vs. 40.5% vs. 58.3%, P < 0.001), congestive heart failure (10.4% vs. 20.6% vs. 25.6%, P < 0.001), chronic kidney disease (11.9% vs. 19.5% vs. 52.9%, P < 0.001), stroke (7.1% vs. 9.3% vs. 11.0%, P = 0.002), and anemia (30.1% vs. 37.5% vs. 58.6%, P < 0.001), The baseline clinical characteristics of patients are reported in Tables 1 and 2.

**Table 1:** Baseline characteristics CAD patients stratified by different uACR group

Characteristics	Overall	uACR < 10 mg/g	10 ≤ uACR < 30 mg/g	uACR ≥ 30 mg/g	P value
	5,960	4,763	681	516	
Age, years	62.8 (10.4)	62.3 (10.3)	64.2 (10.4)	64.8 (10.8)	< 0.001
Female	1435 (24.1)	1088 (22.8)	180 (26.4)	167 (32.4)	< 0.001
Smoking history	1379 (23.8)	1132 (24.4)	154 (23.2)	93 (18.5)	0.011
Acute myocardial infarction	958 (16.1)	696 (14.6)	141 (20.7)	121 (23.4)	< 0.001
Hypertension	3639 (61.1)	2774 (58.2)	446 (65.5)	419 (81.2)	< 0.001
Type 2 diabetes mellitus	1888 (31.7)	1311 (27.5)	276 (40.5)	301 (58.3)	< 0.001
Congestive heart failure	768 (12.9)	496 (10.4)	140 (20.6)	132 (25.6)	< 0.001
Chronic kidney disease	971 (16.3)	565 (11.9)	133 (19.5)	273 (52.9)	< 0.001
Atrial fibrillation	256 (4.3)	185 (3.9)	41 (6.0)	30 (5.8)	0.008
Stroke	460 (7.7)	340 (7.1)	63 (9.3)	57 (11.0)	0.002
Hyperlipemia	4021 (67.5)	3189 (67.0)	454 (66.7)	378 (73.3)	0.013
Anemia	1941 (33.5)	1399 (30.1)	247 (37.5)	295 (58.6)	< 0.001
PCI	4397 (73.8)	3480 (73.1)	509 (74.7)	408 (79.1)	0.011
uACR, mg/g	14.3 (48.5)	2.0 (2.2)	16.0 (5.3)	125.9 (115.4)	< 0.001
eGFR, mL/min/1.73m^2^	92.7 (46.8)	95.0 (43.8)	91.0 (48.0)	74.3 (64.8)	< 0.001
Hemoglobin, g/L	133.2 (17.2)	134.7 (15.9)	132.2 (17.0)	120.5 (23.4)	< 0.001
LDLC, mmol/L	2.8 (0.9)	2.8 (0.9)	2.9 (0.9)	3.0 (1.1)	< 0.001
HDLC, mmol/L	1.0 (0.2)	1.0 (0.2)	1.0 (0.3)	1.0 (0.3)	0.200
LVEF, n(%)	58.8 (11.9)	59.7 (11.3)	56.6 (13.3)	54.0 (13.5)	< 0.001
HbA1c, n(%)	6.5 (1.4)	6.3 (1.3)	6.7 (1.6)	7.2 (1.8)	< 0.001
ACEI/ARB	3961 (66.9)	3202 (67.5)	450 (66.7)	309 (61.3)	0.019
β-blocker	4937 (83.4)	3943 (83.1)	577 (85.5)	417 (82.7)	0.282
Statins	5030 (84.9)	4097 (86.4)	523 (77.5)	410 (81.3)	< 0.001
Spironolactone	682 (11.5)	435 (9.2)	109 (16.1)	138 (27.4)	< 0.001
Dual antiplatelet therapy	4256 (71.9)	3420 (72.1)	457 (67.7)	379 (75.2)	0.013

Values are mean ± SD, n (%), or median (IQR).

PCI: percutaneous coronary intervention; uACR: urine albumin creatinine ratio; eGFR: estimated glomerular filtration rate; LDLC: low density lipoprotein cholesterol; HDLC: high density lipoprotein cholesterol; HbAlc: glycosylated hemoglobin; ACEI: angiotensin-converting enzyme inhibitors; ARB: angiotensin receptor blockers

**Table 2:** Baseline characteristics CAD patients stratified by T2DM

Characteristics	Overall	without T2DM	T2DM	P value
	5,960	4,072	1,888	
Age, years	62.8 (10.4)	62.6 (10.6)	63.2 (9.9)	0.027
Female	1435 (24.1)	908 (22.3)	527 (27.9)	< 0.001
Smoking history	1379 (23.8)	1007 (25.4)	372 (20.3)	< 0.001
Acute myocardial infarction	958 (16.1)	640 (15.7)	318 (16.8)	0.288
Hypertension	3639 (61.1)	2327 (57.1)	1312 (69.5)	< 0.001
Type 2 diabetes mellitus	1888 (31.7)	0 (0.0)	1888 (100.0)	< 0.001
Congestive heart failure	768 (12.9)	478 (11.7)	290 (15.4)	< 0.001
Chronic kidney disease	971 (16.3)	540 (13.3)	431 (22.8)	< 0.001
Atrial fibrillation	256 (4.3)	168 (4.1)	88 (4.7)	0.379
Stroke	460 (7.7)	277 (6.8)	183 (9.7)	< 0.001
Hyperlipemia	4021 (67.5)	2620 (64.3)	1401 (74.2)	< 0.001
Anemia	1941 (33.5)	1197 (30.2)	744 (40.5)	< 0.001
PCI	4397 (73.8)	2918 (71.7)	1479 (78.3)	< 0.001
uACR, mg/g	14.3 (48.5)	8.7 (33.1)	26.5 (69.7)	< 0.001
eGFR, mL/min/1.73m2	92.7 (46.8)	93.4 (44.6)	91.4 (51.2)	0.126
Hemoglobin, g/L	133.2 (17.2)	134.7 (16.3)	129.8 (18.8)	< 0.001
LDLC, mmol/L	2.8 (0.9)	2.9 (0.9)	2.8 (0.9)	< 0.001
HDLC, mmol/L	1.0 (0.2)	1.0 (0.2)	0.9 (0.2)	< 0.001
LVEF, n(%)	58.8 (11.9)	59.5 (11.5)	57.3 (12.3)	< 0.001
HbA1c, n(%)	6.5 (1.4)	5.8 (0.6)	7.8 (1.6)	< 0.001
ACEI/ARB	3961 (66.9)	2701 (66.7)	1260 (67.3)	0.623
β-blocker	4937 (83.4)	3338 (82.4)	1599 (85.5)	0.004
Statins	5030 (84.9)	3434 (84.7)	1596 (85.3)	0.607
Spironolactone	682 (11.5)	409 (10.1)	273 (14.6)	< 0.001
Dual antiplatelet therapy	4256 (71.9)	2869 (70.8)	1387 (74.1)	0.009

Values are mean ± SD, n (%), or median (IQR).

PCI: percutaneous coronary intervention; uACR: urine albumin creatinine ratio; eGFR: estimated glomerular filtration rate; LDLC: low density lipoprotein cholesterol; HDLC: high density lipoprotein cholesterol; HbAlc: glycosylated hemoglobin; ACEI: angiotensin-converting enzyme inhibitors; ARB: angiotensin receptor blockers

### The association of the baseline uACR with the risk of the mortality

**Fig. 2:**
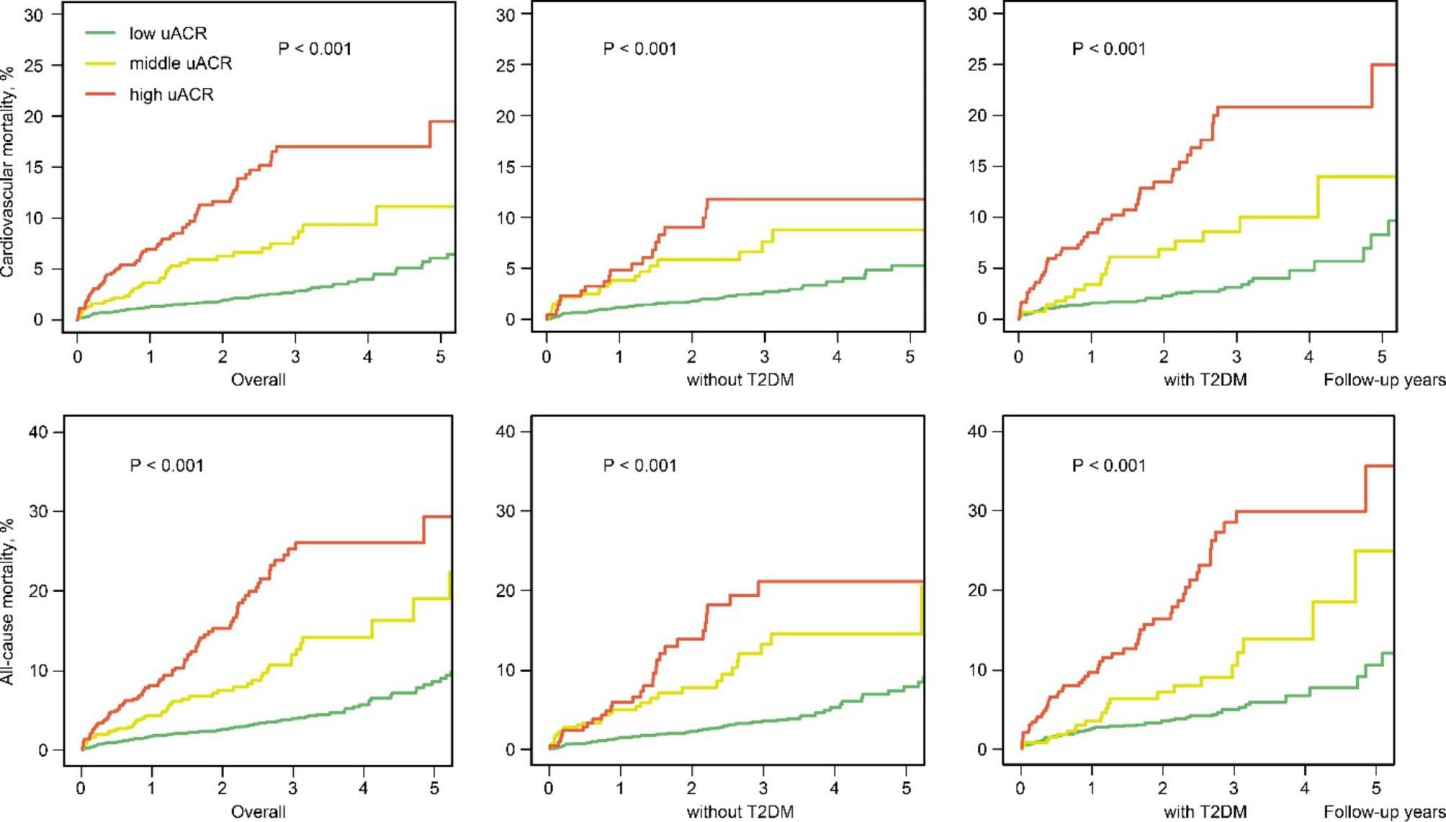
Cumulative hazard cardiovascular and all-cause mortality among CAD patients and different glycemic status (with or without T2DM) Abbreviation: T2DM: type 2 diabetes mellitus; uACR: urinary albumin creatinine ratio. Low uACR: uACR < 10 mg/g; Middle uACR: 10 ≤ uACR < 30 mg/g; High uACR: uACR ≥ 30 mg/g

During the median follow-up of 2.2 [1.2–3.1] years, 310 patients (5.2%) died, of which 236 patients (4.0%) died of cardiovascular disease. Patients with the increase of uACR level had higher all-cause mortality (3.5% vs. 8.4% vs. 17.1%, P < 0.001) and cardiovascular mortality (2.5% vs. 6.6% vs. 13.6%, P < 0.001). (Fig. 2) After adjusting for confounders, patients with increased uACR level had high risk of cardiovascular mortality (middle uACR: HR, 2.32; 95%CI [1.58–3.41], P < 0.001; high uACR: HR, 3.22; 95%CI [2.23–4.66], P < 0.001) as well as all-cause mortality (middle uACR: HR, 2.05; 95%CI [1.46–2.89], P < 0.001; high uACR: HR, 3.08; 95%CI [2.23–4.24], P < 0.001) than those with low uACR level.

**Fig. 3:**
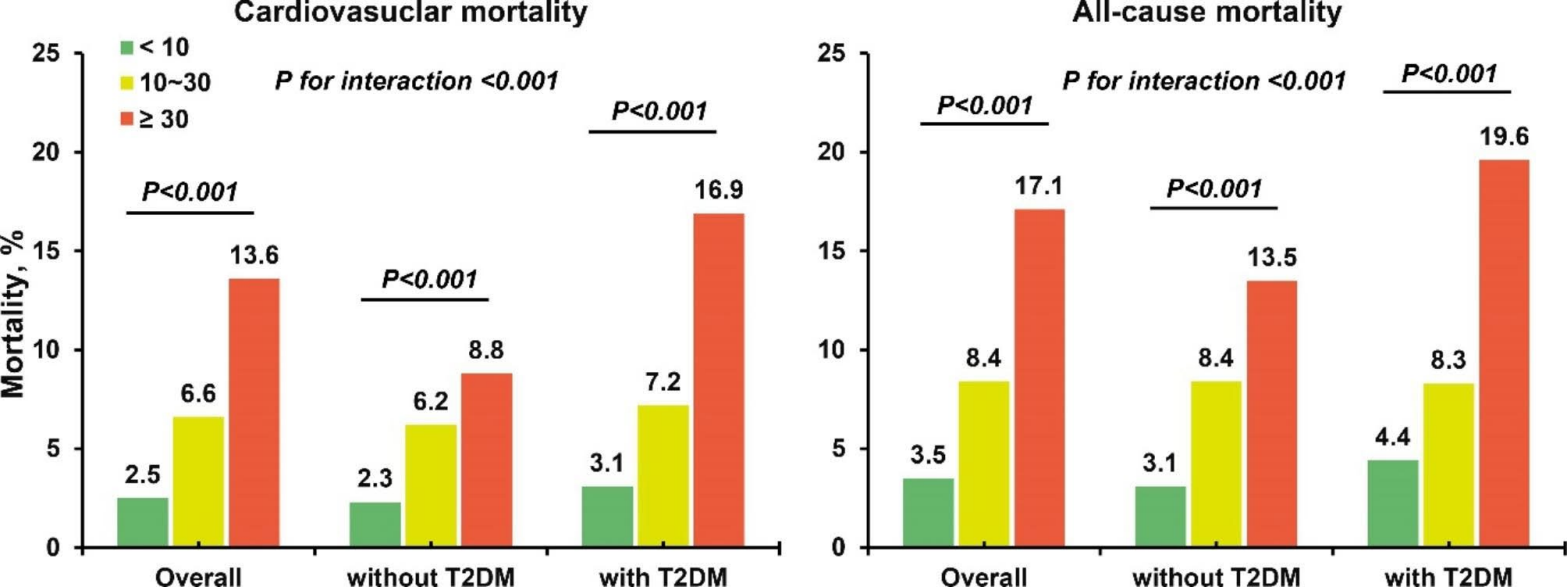
Cardiovascular and all-cause mortality in different uACR level among CAD patients and different glycemic status (with or without T2DM) and the interaction effect between uACR level and different glycemic status Abbreviation: T2DM: type 2 diabetes mellitus; uACR: urinary albumin creatinine ratio. UACR were stratified into < 10 mg/g; 10 ~ 30 mg/g; ≥ 30 mg/g. P for interaction analysed for uACR group and glycemic status (with T2DM or not) and adjusted for age, gender, acute myocardial infarction, hypertension, chronic kidney disease, congestive heart failure, anemia, T2DM, low density lipoprotein cholesterol, and high density lipoprotein cholesterol

**Fig. 4:**
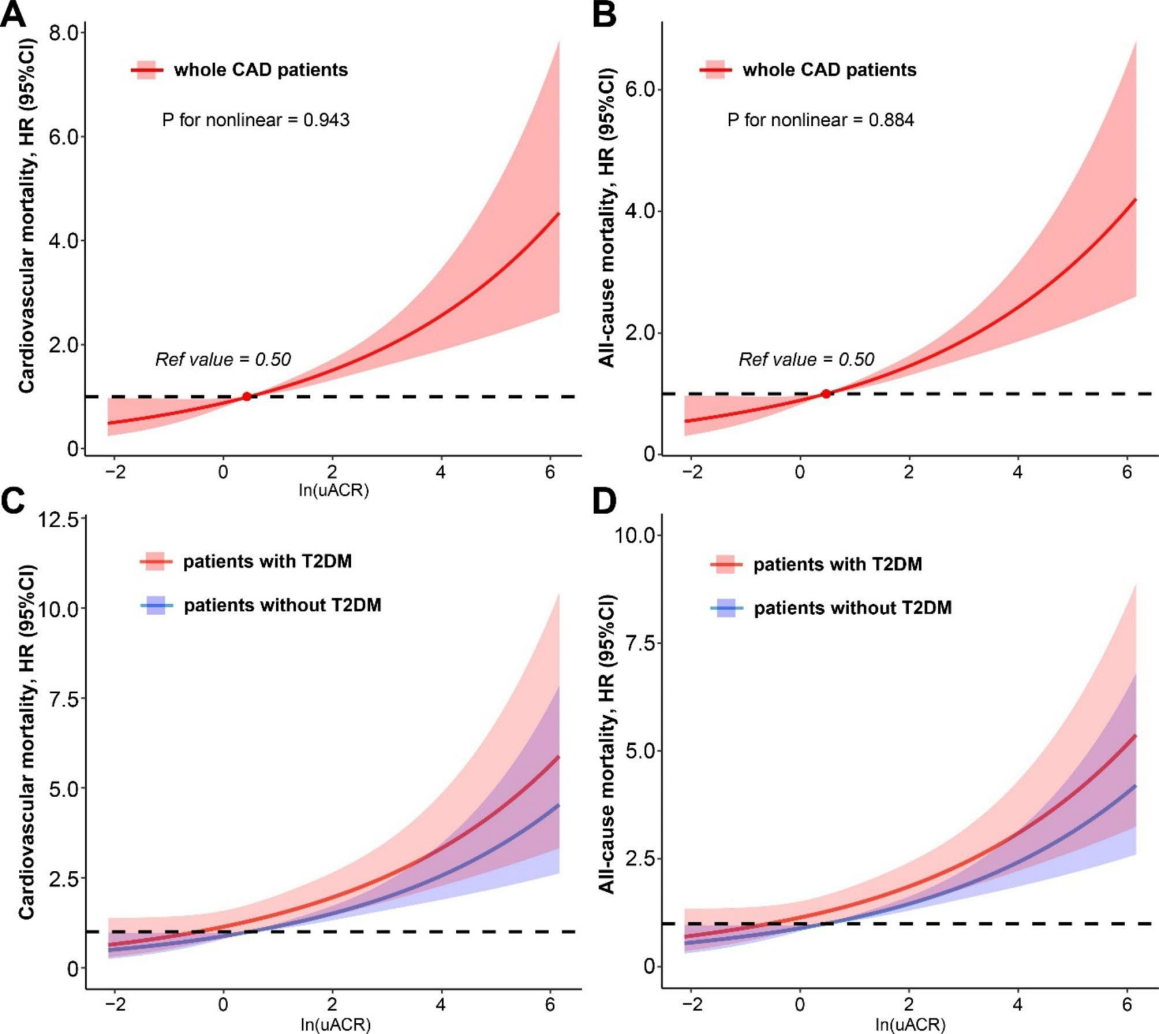
Restricted cubic spline illustrates the relationship between uACR and the risk of cardiovascular and all-cause mortality Abbreviation: T2DM: type 2 diabetes mellitus; uACR: urinary albumin creatinine ratio **A** and **B**: adjusted for age, gender, smoking history, acute myocardial infarction, hypertension, chronic kidney disease, congestive heart failure, anemia, T2DM, low density lipoprotein cholesterol, high density lipoprotein cholesterol and ACEI/ARB **C** and **D**: adjusted for age, gender, smoking history, acute myocardial infarction, hypertension, chronic kidney disease, congestive heart failure, anemia, low density lipoprotein cholesterol, high density lipoprotein cholesterol and ACEI/ARB

When considering patients glycemic status, compared with patients with low uACR, among those with T2DM, patients with uACR level increased still had high risk of cardiovascular mortality (middle uACR: HR, 2.49; 95%CI [1.39–4.46], P < 0.05; high uACR: HR, 3.98; 95%CI [2.44–6.51], P < 0.001) and all-cause mortality (middle uACR: HR, 1.92; 95%CI [1.14–3.23], P < 0.05; high uACR: HR, 3.04; 95%CI [1.98–4.68], P < 0.001). Similarly, patients without T2DM also had higher risk of cardiovascular mortality (middle uACR: HR, 2.33; 95%CI [1.39–3.91], P < 0.05; high uACR: HR, 2.34; 95%CI [1.27–4.18], P < 0.05) and all-cause mortality: (middle uACR: HR, 2.31 [95% CI 1.47–3.63], P < 0.001; high uACR: HR, 2.90 [95% CI 1.78–4.73], P < 0.001). (Table 3) Furthermore, there was interaction effect between glycemic status and uACR level in cardiovascular and all-cause mortality (both P for interaction < 0.001). (Fig. 3) The restricted cubic splines curves showed a linear relationship between ln(uACR) and the risks of cardiovascular and all-cause mortality in the whole CAD patients. (Fig. 4)

**Table 3:** Multivariate risk regression of different groups

Groups	Events, n(%)	HR (95% CI)		
		Low uACR	Middle uACR	High uACR
Whole CAD patients^#^				
Cardiovascular mortality	236 (4.0)	Ref	2.32 (1.58, 3.41)**	3.22 (2.23, 4.66)**
All-cause mortality	310 (5.2)	Ref	2.05 (1.46, 2.89)**	3.08 (2.23, 4.24)**
Without T2DM^$^				
Cardiovascular mortality	124 (3.0)	Ref	2.33 (1.39, 3.91)*	2.34 (1.27, 4.18)*
All-cause mortality	170 (4.2)	Ref	2.31 (1.47, 3.63)**	2.90 (1.78, 4.73)**
With T2DM^$^				
Cardiovascular mortality	112 (5.9)	Ref	2.49 (1.39, 4.46)*	3.98 (2.44, 6.51)**
All-cause mortality	140 (7.4)	Ref	1.92 (1.14, 3.23)*	3.04 (1.98, 4.68)**

**: P < 0.001; *: P < 0.05

Low uACR: uACR < 10 mg/g; Middle uACR: 10 ≤ uACR < 30 mg/g; High uACR: uACR ≥ 30 mg/g

#: Multivariate risk regression adjusted for age, gender, smoking history, acute myocardial infarction, hypertension, chronic kidney disease, congestive heart failure, anemia, T2DM, low density lipoprotein cholesterol, high density lipoprotein cholesterol and ACEI/ARB.

$: Multivariate risk regression adjusted for age, gender, smoking history, acute myocardial infarction, hypertension, chronic kidney disease, congestive heart failure, anemia, low density lipoprotein cholesterol, high density lipoprotein cholesterol and ACEI/ARB.

## Discussion

In this study, we investigate whether mildly elevated uACR can increase the risk of mortality in CAD patients, and whether this risk will be influenced by diabetes status. Our findings indicate that with the elevation of uACR level, the incidence and risk of cardiovascular mortality and all-cause mortality increased among CAD patients, and this risk is more significant in those with T2DM. Even if the uACR level is within the normal range recommended by guidelines, its elevation still increases nearly 1.5-fold risk of mortality among CAD patients.

In clinical practice, uACR, a simple and easy to obtain index, has been recommended for the evaluation in patients with diabetes, hypertension and chronic kidney disease [18, 19]. We evaluated the predictive value of elevated uACR levels on the cardiovascular and all-cause mortality among patients with CAD, and found that moderately elevated uACR levels increased the risk of mortality by 1 to 1.5 times, while severe elevated uACR would increase the risk of mortality by over 2 times. Similar to our study, Mok et al. conducted a study with 838 patients with atherosclerotic cardiovascular disease in a study, and found that ACR ≥ 30 mg/g increased the risk of adverse outcomes by nearly 50%, which was an independent predictor of poor prognosis and had potential value for secondary prevention for patients with atherosclerotic cardiovascular disease [20]. However, atherosclerotic cardiovascular disease was not classified in their study, and our data further indicate that even a low-level elevation in uACR (10–30 mg/g) are associated with increased cardiovascular and all-cause mortality risk than patients with uACR < 10 mg/g. Notably, Gerstein et al. have reported that any degree of albuminuria (any increase of uACR) is a risk factor for cardiovascular events in subjects with or without DM, but the patients enrolled in this study was lower risk than in our study as well (aged 55 years or more with a history of CV disease) [21]. Therefore, uACR, as an easily available and valuable prognostic indicator, deserves attention among CAD patients.

Our results suggest that the risk of mortality increases in CAD patients with or without T2DM as uACR increases. We find that CAD patients with DM are older and had more cardiovascular risk factors, which is similar with current studies [22]. Urinary protein excretion reflects systemic endothelial leakiness, which increases albumin permeability during vascular endothelial injury or compression, resulting in vascular lesions [23]. Persistent micro- or macroalbuminuria are contemporary considered as the markers of generalized vascular endothelial damage[4, 24]. Endothelial damage further leads to high uACR levels with an increase in the severity of CVD and results in much greater atherosclerotic burden. In addition, previous study has shown that endothelial injury mechanisms promote disease progression in patients with cardiovascular risk factors such as diabetes [25]. A key role is played by a polysaccharide gel called glycocalyx, which acts as a barrier against albumin filtration, endothelial cell activation and glycocalyx degradation, leading to atherosclerosis, which lead to cardiovascular disease together [26, 27]. Diabetes further worsened the prognosis of patients with CAD, which was consistent with the results of previous reports. Increased uACR level among DM patients also means that endothelial dysfunction increases the overproduction of reactive oxygen species, which will activate platelet activity and stimulate vascular smooth muscle proliferation. It accelerates the occurrence and progression of CAD, resulting in the deterioration of the prognosis of patients [28, 29]. Therefore, we believe that the elevation of uACR may be closely related to the occurrence and development of CAD, and diabetes can further deteriorate this process.

Furthermore, our study observe that increased uACR has a more significant impact on all-cause and cardiovascular mortality in patients with T2DM than in patients without T2DM, which may warrant a more comprehensive evaluation in patients with T2DM. Our study suggests that cardiologists may include uACR as a predictor of prognosis in patients with CAD, especially when combined with T2DM, and elevated uACR increases the risk of all-cause and cardiovascular mortality, even within the normal range recommended by guidelines. For patients with CAD, it is also necessary to receive uACR assessment early after discharge to identify high-risk patients and control their urinary protein levels using SGLT-2, ACE-inhibitors and statins [30–32]. More prospective studies are needed in the future to explore the diagnostic strategy and treatment of this high-risk population can be further included in the guidelines in the future, which will benefit more patients.

There were several limitations in this study. Firstly, this study has limitations inherent in retrospective analysis, including data incompleteness, such as duration of diabetes or body mass index, cannot be entirely ruled out. Secondly, our research is a retrospective study, and uACR is only measured once, which may be affected by multiple influences and lead to inaccurate results. Thirdly, we do not consider the effect of drugs. Some cardiovascular and diabetes drugs commonly used in the patient in this study, such as ACE-inhibitors and SGLT-2 (discharge medicine), can also reduce uACR, which is not corrected in this study. Fourthly, Due to the nature of the observational study, the observation period is short compared to the study period, which may have introduced attrition bias. Fifthly, the measurement of proteinuria may be bias due to the time, personnel and other factors, but our participating centers use the same measurement method and the central laboratory regularly performed spot checks on urine samples to ensure the accuracy of the data. Finally, our study endpoint is limited to mortality and do not examine the occurrence of other events. But our data comes from the real world, and it directly reflects patient outcomes.

## Conclusions

In our study, with the elevation of uACR level, the risk of cardiovascular mortality and all-cause mortality increase among CAD patients, and this risk is more significant in those with T2DM. Even if the uACR level increases mildly within the normal range recommended by guidelines, its elevation still increases nearly 1.5-fold risk of mortality among CAD patients.

### Electronic supplementary material

Below is the link to the electronic supplementary material.

Supplementary Material 1

## Data Availability

The datasets used and/or analysed during the current study are available from the corresponding author on reasonable request.

## Abbreviations

CAD: Coronary artery disease
MACE: Major adverse cardiovascular events
T2DM: Type 2 diabetes mellitus
UACR: Urine albumin creatinine ratio
