# Supplementary material for: Elevated urine albumin creatinine ratio increases cardiovascular mortality in coronary artery disease patients with or without type 2 diabetes mellitus: a multicenter retrospective study
Source: Cardiovasc Diabetol. 2023 Aug 10;22:203. doi: 10.1186/s12933-023-01907-3 (PMC10416404; doi:10.1186/s12933-023-01907-3)
Supplement: Supplementary file 1 — Supplementary Material 1 [file 12933_2023_1907_MOESM1_ESM.docx]

Supplement figure 1: Restricted cubic spline illustrates the relationship between uACR and the risk of cardiovascular and all-cause mortality among CAD patients combined with CHF


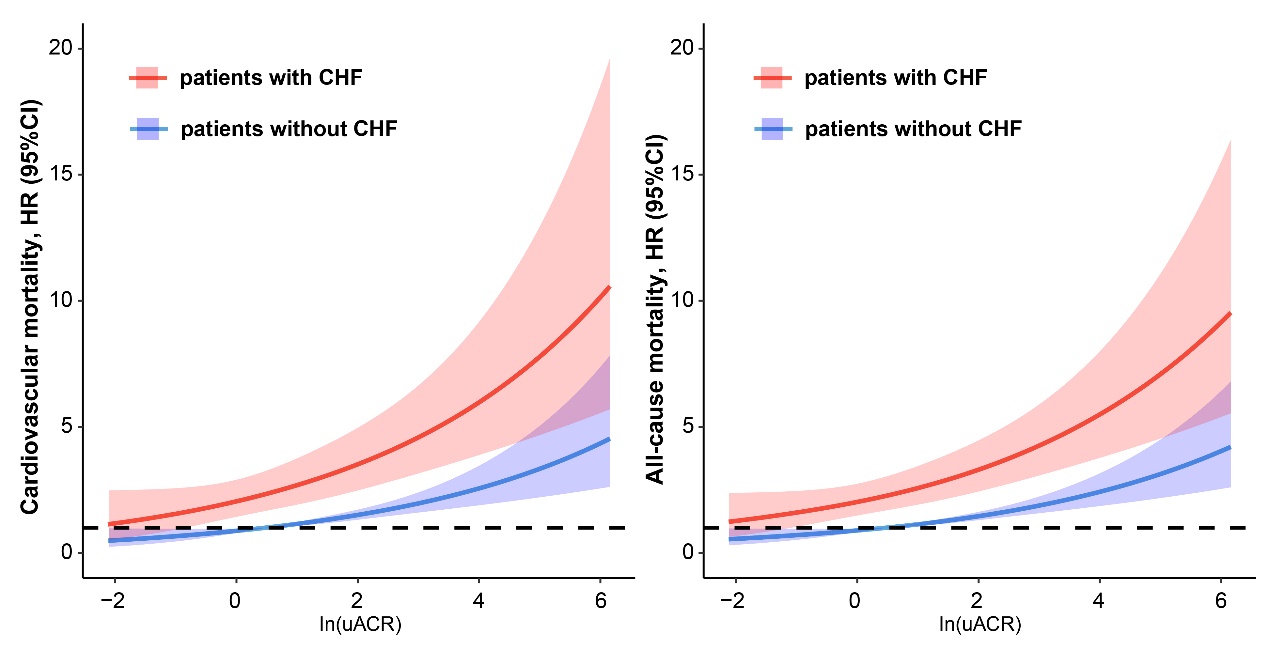


Abbreviation: uACR: urinary albumin creatinine ratio; CHF: congestive heart failure

Adjusted for age, gender, smoking history, acute myocardial infarction, hypertension, chronic kidney disease, anemia, type 2 diabetes mellitus, low density lipoprotein cholesterol, high density lipoprotein cholesterol and ACEI/ARB.
